# Enhanced Corrosion Resistance of Layered Double Hydroxide Films on Mg Alloy: The Key Role of Cationic Surfactant

**DOI:** 10.3390/ma15062028

**Published:** 2022-03-09

**Authors:** Qiuxiang Yang, Mohammad Tabish, Jingbao Wang, Jingmao Zhao

**Affiliations:** 1 School of Materials Science and Engineering, Beijing University of Chemical Technology, Beijing 100029, China; 18810130903@163.com (Q.Y.); tabish.5000@buct.edu.cn (M.T.); 2020400110@mail.buct.edu.cn (J.W.); 2 Beijing Key Laboratory of Electrochemical Process and Technology for Materials, Beijing 100029, China

**Keywords:** magnesium alloy, cationic surfactant, layered double hydroxide film, corrosion resistance

## Abstract

In this study, dense anticorrosion magnesium–aluminum layered double hydroxide (MgAl-LDH) films were prepared for the first time by introducing a cationic surfactant tetradecyltrimethyl ammonium bromide (TTAB) in the process of in situ hydrothermal synthesis of Mg-Al LDH films on an AZ31 magnesium alloy. Results of XRD, FTIR, and SEM confirmed that TTAB forms the MgAl-LDH-TTAB, although TTAB cannot enter into LDH layers, and MgAl-LDH-TTAB powders are much smaller and more homogenous than MgAl-CO_3_^2−^-LDH powders. Results of SEM, EDS, mapping, and XPS confirmed that TTAB forms the MgAl-LDH-TTAB films and endows LDH films with denser structure, which provides films with better shielding efficiency. Results of potentiodynamic polarization curves (PDP) and electrochemical impedance spectroscopy (EIS) confirmed that MgAl-LDH-TTAB_x g_ films have better corrosion resistance than an MgAl-CO_3_^2−^-LDH film. The corrosion current density (*i_corr_*) of the MgAl-LDH-TTAB_0.35 g_ film in 3.5 wt.% NaCl solution was reduced to 1.09 × 10^−8^ A.cm^−2^ and the |Z|_f = 0.05 Hz_ value was increased to 4.48 × 10^5^ Ω·cm^2^. Moreover, the increasing concentration of TTAB in MgAl-LDH-TTAB_x g_ (x = 0.025, 0.05, 0.1, 0.2 and 0.35) provided denser outer layer LDH films and thereby increased the corrosion resistance of the AZ31 Mg alloy. Additionally, the |Z|_f = 0.05 Hz_ values of the MgAl-LDH-TTAB_0.35 g_ film still remained at 10^5^ Ω·cm^2^ after being immersed in 3.5 wt.% NaCl solution for 168 h, implying the good long-term corrosion resistance of MgAl-LDH-TTAB_x g_ films. Therefore, introducing cationic surfactant in the process of in situ hydrothermal synthesis can be seen as a novel approach to creating efficient anticorrosion LDH films for Mg alloys.

## 1. Introduction

Magnesium and its alloys have gained a lot of interest over the years owing to their many advantages, such as high specific strength, high thermal conductivity, excellent machinability, and ease of recycling. They are, nevertheless, extremely caustic, particularly in corrosive environments containing Cl^−^. Because of their low corrosion resistance, they are severely limited in their uses in aerospace, biomedical engineering, automotive, and a variety of other areas. Several anticorrosion approaches for Mg alloys have been established so far, including organic coating [1,2], chemical conversion coating [3,4], surface chemical modification [5,6], micro-arc oxidation (MAO) [7,8], as well as an inhibitor [9,10]. In situ generated layered double hydroxide (LDH) coatings on Mg alloys have recently piqued the interest of many researchers owing to its outstanding anticorrosion performance and strong adherence to the substrate [11]. LDH films have high corrosion resistance due to three factors: (i) the LDH film acts as a strong physical barrier to corrosive media such as water molecules and aggressive anions, (ii) LDH can capture aggressive anions, such as Cl^−^, due to their unique interlayer anion exchangeability, and (iii) LDH with corrosion inhibitor intercalation can release an inhibitor [12,13,14,15].

In the past years, a variety of treatment techniques have been used to improve the corrosion resistance of the LDH film. Several researchers developed LDH films with corrosion inhibitor intercalation to enhance their corrosion resistance, such as aspartic acid [12], 8-hydroxyquinoline [16], and phenylphosphonic acid [17]. Some researchers also utilized low surface energy substances, such as aliphatic carboxylic acid [13] and fluoroalkyl silane [18], to obtain superhydrophobic LDH films with improved corrosion resistance. Additionally, the formation of dense LDH films on the Mg alloy surface is another effective method for obtaining LDH films with a high corrosion resistance. Li [19] used a thiophene derivative to produce highly corrosion-resistant LDH coatings with no big holes or fractures on the surface of a magnesium alloy. Ahsan Iqbal [20] proposed that more compact and dense LDH films can be prepared at 100 °C for an 18 h reaction time with improved corrosion resistance. Zhou [21] created a ZnAl-LDHs film with a finer and denser structure on the surface of Al alloys by adding lanthanum ions, and the denser nanosheets of ZnLaAl-LDH contributed to the improvement of anticorrosion performance.

In order to synthesize nanostructured LDH, different surfactants, including both anionic surfactant (sodium dodecyl sulfate, SDS) [22,23] and cationic surfactant (cetyltrimethylammonium bromide, CTAB) [22,24], have been used as soft and self-assembled templates to control the size and shape of nanoparticles. The MgAl-LDH-CTAB platelet has been found to be much smaller than the MgAl-CO_3_-LDH platelet [22]. Many studies have shown that surfactants may significantly enhance the characteristics of electrodeposited nanocomposite coatings on metal surfaces by contributing to the formation of a homogeneous and dense coating [25,26]. However, no studies have been reported to create in situ synthesis anticorrosion LDH films on metal surfaces with a surfactant. Therefore, it is essential to explore the effect of surfactants on the synthesis of LDH corrosion-resistant films. It is also important to explore the effect of surfactants on the synthesis of LDH corrosion-resistant coatings. Anionic surfactants may penetrate an LDH layer and modify the LDH laminate spacing through an ion exchange [22], but cationic surfactants will not have such effects [22]; only the cationic surfactant was used in this study. In addition, according to previous studies, the metal ions can release from the Mg substrate in hydrothermal synthesis and participate in the in situ formation of LDH film, which led to the high adhesion between the LDH film and the substrate [27,28].

In this study, high corrosion resistant MgAl-LDH films were produced on AZ31 Mg alloys via an in situ hydrothermal method with the cationic surfactant tetradecyltrimethyl bromide (TTAB, CH_3_(CH_2_)_13_N(Br)(CH_3_)_3_) to further enhance the protection ability of LDH films and provide a better corrosion resistance to the Mg substrate against an aggressive electrolyte. Scanning electron microscopy (SEM), Fourier transform infrared spectroscopy (FTIR), and X-ray diffraction (XRD) examined the impact of TTAB on the microstructure and chemistry of LDH particles. The findings have shown the success of the synthesis of MgAl-LDH-TTAB, and of the smaller and more homogeneous particles of MgAl-CO_3_^2−^-LDH particles. LDH films were investigated using X-ray photoelectron spectroscopy (XPS) and SEM to evaluate the chemical compositions and surface morphology. The findings indicated that the films of MgAl -LDH-TTAB_x g_ are more consistent and compact than the MgAl-CO_3_^2−^-LDH film. The corrosion resistance of LDH films was studied by electrochemical methods (PDP and EIS). A change in coating morphology is primarily attributable to the excellent resistance to corrosion in MgAl-LDH-TTABxg films. The corrosion current density of the Mg alloy was decreased to 1 × 10^−8^A·cm^−2^. Therefore, utilization of cationic surfactant to synthesis the LDH films improves corrosion efficiency.

## 2. Materials and Methods

### 2.1. Materials and Chemicals

AZ31 Mg alloy (3 wt.% Al, 1 wt.% Zn, 0.4 wt.% Mn, balance Mg) sheets with a dimension of 25 × 25 × 2 mm were chosen in this study. All chemicals, including sodium carbonate, magnesium nitrate, aluminum nitrate, tetradecyltrimethylammonium bromide (TTAB), sodium chloride, and sodium hydroxide, were obtained from Sinopharm Chemical Reagent Co. Ltd., Beijing, China. with analytical grade. Deionized water (DI) was utilized to prepare all of the solutions.

### 2.2. In Situ Fabrications of MgAl-LDH-TTAB_x g_ and MgAl-CO_3_^2−^-LDH Films

**Figure 1:**
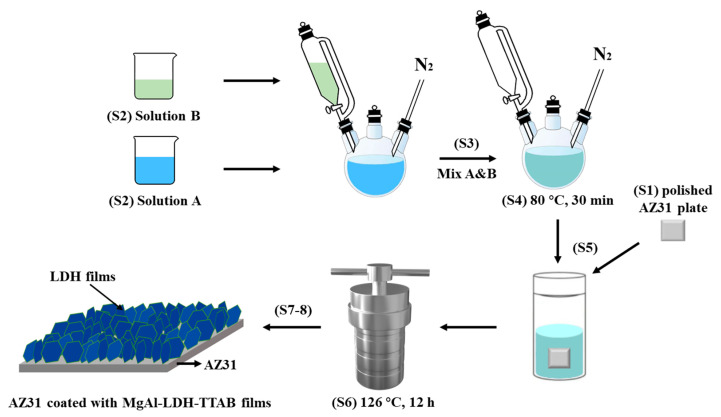
Schematic illustration of the preparation of MgAl-LDH-TTAB films.

According to reported methods, MgAl-LDH-TTAB_x g_ films were prepared via in situ hydrothermal synthesis with surfactant TTAB [29]. The in situ preparation refers to putting AZ31 substrate and LDH precursor solution into the autoclave (Yuze Industrial Technology Co., Ltd., Jining, China), and when they make contact, metal ions are released from the Mg substrate and participate in the formation of LDH film on the surface of the Mg alloy. As shown in Figure 1, the detailed synthesis process can be divided into the following steps. In step 1 (S1), the surface of the AZ31 plates was polished with 800, 1200, 1500, and 2000# grit SiC abrasive sheets (Kuangyu Metal Co., Ltd., Dongguan, China), cleaned in ethanol ultrasonically and dried with warm air. In step 2 (S2), solution A with a Mg^2+^/Al^3+^ molar ratio of 3.0 was obtained by dissolving 6 g magnesium nitrate and 3 g aluminum nitrate dissolved in 120 mL of DI water in a three-neck flask, and solution B was separately obtained by dissolving 0.025/0.05/0.1/0.2/0.35 g TTAB and 2 g NaOH in 80 mL DI water at 40 °C. In Step 3 (S3), the precursor solution was made by the progressive addition of solution B to solution A at 80 °C with continuous stirring, and the pH of the precursor solution was adjusted to 10.5 with NaOH solution (2 mol/L). In step 4 (S4), the combined solution was allowed to react for 30 min under continuous stirring. In step 5 (S5), the polished AZ31 plates were put into the Teflon-lined autoclave with the combined solution prepared in S4. In step 6 (S6), the autoclave was heated for the hydrothermal reaction at 126 °C for 12 h in an oven (Dute Scientific Instrument Co., Ltd., Shanghai, China) [13]. In step 7 (S7), the Teflon-lined autoclave (Yuze Industrial Technology Co., Ltd., Jining, China) was taken out from the oven and then cooled down to room temperature. In step 8 (S8), AZ31 samples were taken out from the Teflon-lined autoclave, washed with DI water and dried with warm air. Finally, AZ31 samples coated with MgAl-LDH-TTAB_x g_ films were obtained. Steps 2, 3 and 4 were carried out in the N_2_ environment in order to prevent carbonate ions from entering LDH interlayers. LDH powder from the LDH precipitates was acquired once the hydrothermal process was completed.

Similarly, AZ31 samples coated with MgAl-CO_3_^2−^-LDH films were synthesized with the major difference in solution B (Step 2), which was obtained from NaOH and Na_2_CO_3_ (the molar ratios of [NaOH]:[Mg^2+^+Al^3+^] = 1.6 and [CO_3_^2−^]:[Al^3+^] = 2.0) dissolved in DI water. Additionally, this experiment was performed in the air instead of N_2_.

The acronyms and the difference of the samples obtained in this section are displayed in Table 1.

### 2.3. Characterization

The crystalline structure of LDH powders from the LDH precipitates after hydrothermal reaction was identified by X-ray diffraction (XRD, Ultima IV, Rigaku-6000, Tokyo, Japan) with K-beta filter Cu Kα radiation (0.15406 nm, 40 kV, 40 mA). A scanning speed of 10deg/min was applied to record the XRD patterns in the 2θ-range degree of 5–90°. FTIR (Bruker, TENSOR-27, Karlsruhe, Germany) spectra with a resolution of 4 cm^−1^ and a scanning range from 4000 to 600 cm^−1^ were acquired to observe the presence of organic functional groups. Scanning electron microscope (SEM, FEI Quanta 200 F, Den Haag, The Netherlands) operating at 30 kV was utilized to examine the morphologies of LDH powders and films. Electron dispersive spectroscopy (EDS, FEI, Den Haag, The Netherlands) coupled to the SEM facility was used to determine the elemental composition of the LDH film. X-ray photoelectron spectroscopy (XPS, Al Kα X-rays, h*v* = 1486.6 eV, Thermo Scientific K-Alpha, Dreieich, Germany) was used to examine the surface chemical compositions of the MgAl-CO_3_^2−^-LDH and MgAl-LDH-TTAB films. All XPS data were fitted with the XPS peak 4.1 software and the background subtraction was carried out by the Tougaard background method. Binding energies were corrected based on the adventitious carbon signal at 284.8 eV in the C 1s spectrum.

The adhesion test of MgAl-LDH-TTAB film on the AZ31 substrate was conducted by a cross-cut tester (QFH-HG600, Shenzhen, China) with tempered steel rule graduated in 1 mm and a 25 mm wide semitransparent pressure-sensitive c provided by the supplier. The experiment was completed according to the experimental method described in the Annual Book of ASTM Standards in accordance with standard ASTM D3359-97 [30]. Optical microscopy (JT-1600B, Shenzhen, China) was used to observe the morphology of the film before and after tape peeling.

### 2.4. Electrochemical Tests

The corrosion behavior of the AZ31 samples covered with various LDH films was evaluated electrochemically with PDP and EIS. All the experiments were conducted on a three-electrode setup with a platinum counter electrode, a saturated calomel reference electrode, and a 0.786 cm^2^ exposed area working electrode. All electrochemical experiments were conducted at room temperature in a 3.5 wt.% NaCl solution. A steady open circuit potential (OCP) was achieved through 30 min of sample immersion before the electrochemical tests. The EIS test was performed at frequencies ranging from 100 kHz to 5 mHz with AC perturbation of 10 mV vs OCP. PDP curves were produced from scanning potential between −500 mV to +500 mV (OCP) with a scanning rate of 0.5 mV·s^−1^.

## 3. Results and Discussion

### 3.1. Characterization of MgAl-CO_3_^2−^-LDH and MgAl-LDH-TTAB Powders by XRD, FTIR and SEM

**Figure 2:**
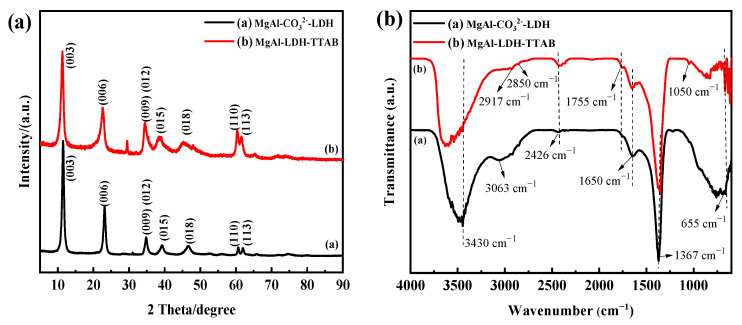
XRD patterns (**a**) and FTIR spectra (**b**) of MgAl-CO_3_^2−^-LDH powders and MgAl-LDH-TTAB powders.

As shown in Figure 2a, the main diffraction peaks of MgAl-CO_3_^2−^-LDH powders were located at 2θ = 11.5, 23.1, 34.8, 39.2, 46.7, 60.6, and 62.1, corresponding to the (003), (006), (009), (012), (015), (018), (110), and (113) planes of a layered hydrotalcite-like material, respectively, and the (009) reflection overlaps with the (012) [31,32]. According to Figure 2a, MgAl-LDH-TTAB powders have similar XRD patterns with MgAl-CO_3_^2−^-LDH powders and the main diffraction peaks were located at 2θ = 11.3, 22.6, 34.4, 39.1, 45.3, 60.3, and 61.5, corresponding to the (003), (006), (009), (012), (015), (018), (110), and (113) planes. The results suggested that MgAl-LDH-TTAB and MgAl-CO_3_^2−^-LDH with hexagonal LDH crystal structure have been successfully synthesized and the cationic surfactant TTAB cannot intercalate the interlayer of the LDH or alter the interlayer spacing of LDH laminates. In the FT-IR spectra of MgAl-CO_3_^2−^-LDH powders and MgAl-LDH-TTAB powders (Figure 2b), the peaks around 3430 cm^−1^ were associated with the –OH stretching band and the peaks around 2850 cm^−1^ and 2917 cm^−1^ were due to carbon–hydrogen (C–H) stretching [33,34,35]. The peaks at the lower wavenumber are associated with bonds between metal and oxygen [36]. Furthermore, the peak of MgAl-LDH-TTAB at 1050 cm^−1^ is ascribed to the C–N bond from TTAB [37,38]. The results substantiate that TTAB forms the MgAl-LDH-TTAB.

**Figure 3:**
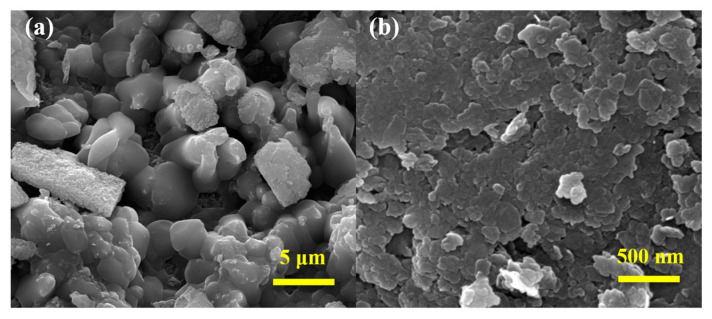
SEM images of (**a**) MgAl-CO_3_^2−^-LDH powders and (**b**) MgAl-LDH-TTAB powders.

Figure 3 shows SEM images of the MgAl-CO_3_^2−^-LDH and MgAl-LDH-TTAB powders with characteristic plate-like and hexagonal LDHs [17,39]. As shown in Figure 3, MgAl-LDH-TTAB powders with a mean particle size of 100–200 nm are much smaller and more homogenous than MgAl-CO_3_^2−^-LDH powders with an average size of 2–4 μm. The results suggest that fine and uniform LDH can be produced by adding cationic surfactant during the process of in situ hydrothermal synthesis.

### 3.2. Characterizations of MgAl-CO_3_^2−^-LDH and MgAl-LDH-TTAB_x g_ Films by SEM, EDS, Mapping, and XPS

**Figure 4:**
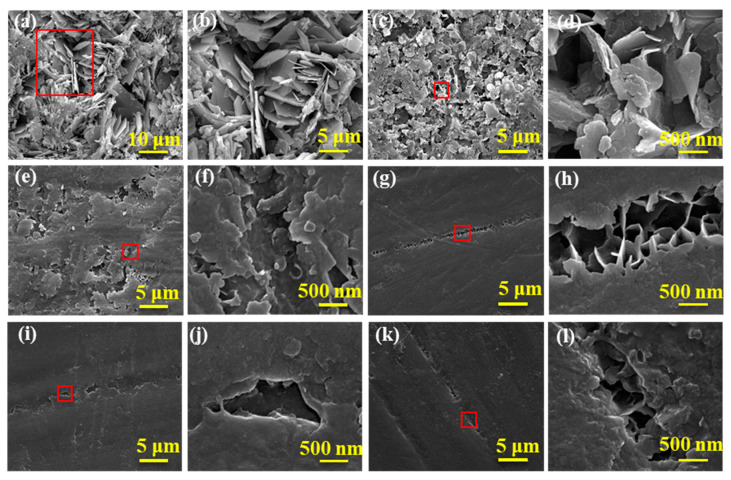
SEM images of LDH films, (**a**,**b**) MgAl-CO_3_^2−^-LDH film, (**c**,**d**) MgAl-LDH-TTAB_0.025 g_ film, (**e**,**f**) MgAl-LDH-TTAB_0.05 g_ film, (**g**,**h**) MgAl-LDH-TTAB_0.1 g_ film, (**i**,**j**) MgAl-LDH-TTAB_0.2 g_ film, (**k**,**l**) MgAl-LDH-TTAB_0.35 g_ film. (**b**,**d**,**f**,**h**,**j**,**l**) are enlarged views of areas marked with red boxes in (**a**,**c**,**e**,**g**,**i**,**k**), respectively.

Figure 4 shows the micrographs of MgAl-CO_3_^2−^-LDH and MgAl-LDH-TTAB_x g_ films with different magnifications. MgAl-CO_3_^2−^-LDH film has a typical blade-like structure, indicating that the metal hydroxide is successfully bound to the interior ions with excellent crystallinity of LDH film [16]. However, as shown in Figure 4a,b, the MgAl-CO_3_^2−^-LDH film has numerous visible pores, resulting in a poor shielding performance of the film. As shown in Figure 4c, the surface morphology of the MgAl-LDH-TTAB_x g_ films becomes much more compact with fewer pores than that of the MgAl-CO_3_^2−^-LDH films, indicating that the MgAl-LDH-TTAB_x g_ films have better shielding performance than the MgAl-CO_3_^2−^-LDH films. Figure 4c,d shows that LDH nanosheets are intersecting perpendicular to the substrate and part of the lamellar LDHs combine to create a floral shape, which is consistent with previously published literature [13,40]. It can readily be observed that the concentration of TTAB has a significant impact on the shape of the resulting film. As shown in Figure 4c,e, at the lower concentrations of TTAB, MgAl-LDH-TTAB_0.025 g/0.05 g_ films still have numerous visible pores. When the concentration of TTAB is increased, as shown in Figure 4g,i,k, MgAl-LDH-TTAB_0.1 g/0.2 g/0.35 g_ films are more compact with fewer pores. Additionally, it is easy to observe in the enlarged views of MgAl-LDH-TTAB_x g_ film that the floral LDH nanosheets were slowly covered by small LDH nanosheets with the increase of TTAB concentration. As a result, MgAl-LDH-TTAB_x g_ films have better shielding performance with the increase of TTAB concentration.

**Figure 5:**
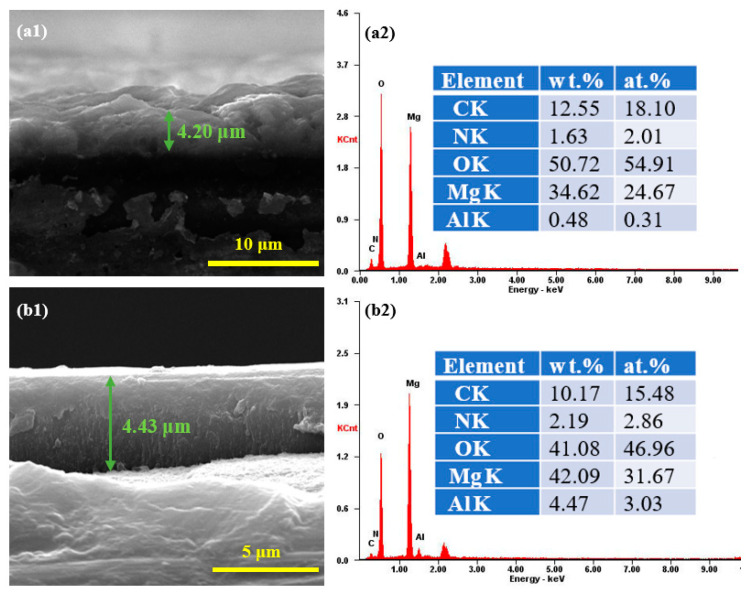
Cross-sections of AZ31 coated with MgAl-CO_3_^2−^-LDH film (**a1**) and MgAl-LDH-TTAB film, (**b1**), and EDS spectra of the surface of AZ31 coated with MgAl-CO_3_^2−^-LDH film (**a2**) and MgAl-LDH-TTAB film (**b2**).

**Figure 6:**
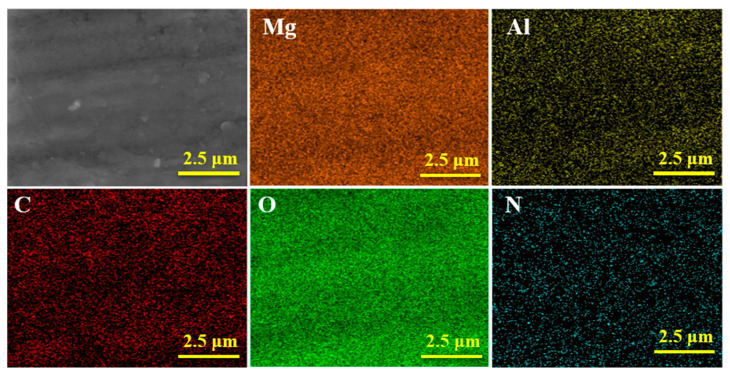
Elemental mappings of the surface of AZ31 coated with MgAl-LDH-TTAB film.

Cross-sectional SEM images of AZ31 coated with MgAl-CO_3_^2−^-LDH and MgAl-LDH-TTAB film are shown in Figure 5(a1,b1), respectively. The MgAl-CO_3_^2−^-LDH film and the MgAl-LDH-TTAB film were approximately 4.20 μm and 4.43 μm in thickness, respectively. The cross-section of the MgAl-LDH-TTAB film revealed a compact hierarchical composite coating structure. EDS spectra of the surface of AZ31 coated with MgAl-CO_3_^2−^-LDH film (a2) and MgAl-LDH-TTAB film (b2) are shown in Figure 5(a2,b2), respectively. The C element of MgAl-CO_3_^2−^-LDH film and MgAl-LDH-TTAB film were from sodium carbonate and TTAB, respectively. Metal cations were from magnesium nitrate, aluminum nitrate, and AZ31 substrate. Results further confirmed that TTAB formed the MgAl-LDH-TTAB. Figure 6 depicts the EDS mapping of the Mg, Al, C, O, and N element in the surface of the MgAl-LDH-TTAB film. These elements uniformly distributed in the MgAl-LDH-TTAB film and confirmed the homogeneity of the MgAl-LDH-TTAB film.

**Figure 7:**
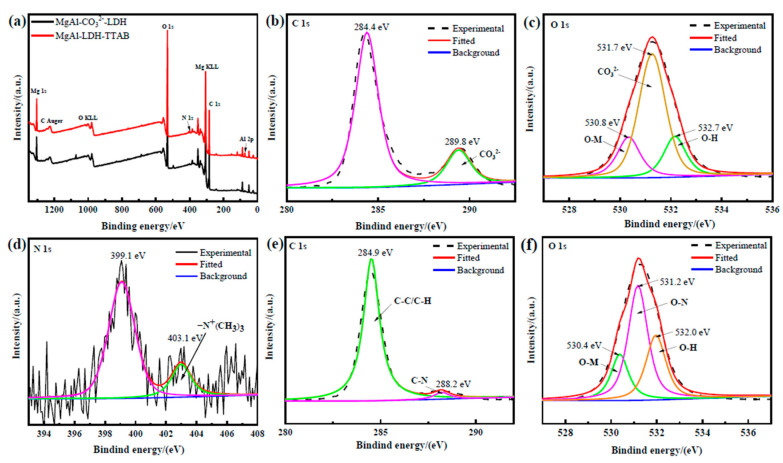
XPS spectra of LDH film, including survey spectra (**a**), C1s and O1s high-resolution spectra (**b**,**c**) of MgAl-CO_3_^2−^-LDH film, and N1s, C1s and O1s high-resolution spectra (**d**–**f**) of MgAl-LDH-TTAB film.

Figure 7 shows the XPS spectra of the surface of the AZ31 Mg substrate coated with MgAl-CO_3_^2−^-LDH film and MgAl-LDH-TTAB film. Figure 7a shows the survey spectra of two LDH-TTAB films. Binding energy peaks of Mg 1s, Al 2p, C 1s, and O 1s was detected from two LDH films, and N 1s was detected from the MgAl-LDH-TTAB film. Figure 7b,c,e,f shows the high-resolution spectra of C1s and O1s of the MgAl-CO_3_^2−^-LDH and MgAl-LDH-TTAB films, respectively. As shown in Figure 7b, the binding energies of 284.4 eV may be HCO_3_^−^ in the interlayer of the LDH [41], and the peak at 289.8 eV represents CO_3_^2−^ of MgAl-CO_3_^2−^-LDH film [16]. The binding energies of 284.9 eV [42] and 288.2 eV [37] illustrated in Figure 7e reflects the C–C/C–H and C–N bond of TTAB in the MgAl-LDH-TTAB film. The peaks at 530.8 eV, 531.7 eV, and 532.7 eV can be ascribed to O–M, CO_3_^2−^, and O–H of the MgAl-CO_3_^2−^-LDH film (Figure 7f), respectively [13,16]. The O–M bond comes from O–Mg or O–Al, and the O–H bond comes from hydroxyl on the LDH and the adsorbed water molecules. The peaks at 530.4 eV, 531.2 eV, and 532.0 eV represent O–M, O–N, and O–H of the MgAl-LDH-TTAB film (Figure 7f), respectively [43]. The N–O bond corresponds to the NO_3_^−^ in LDH [44]. Figure 7d shows N1s spectra of the MgAl-LDH-TTAB film. The peak at 403.1 eV represents -N^+^(CH_3_)_3_ in the TTAB and the peak at 399.1 eV can be attributed to NO_3_^−^ in LDH [45,46]. The results are consistent with the results of EDS and mapping and evince that TTAB successfully formed the MgAl-LDH-TTAB film.

### 3.3. Adhesion Test of MgAl-LDH-TTAB Films

**Figure 8:**
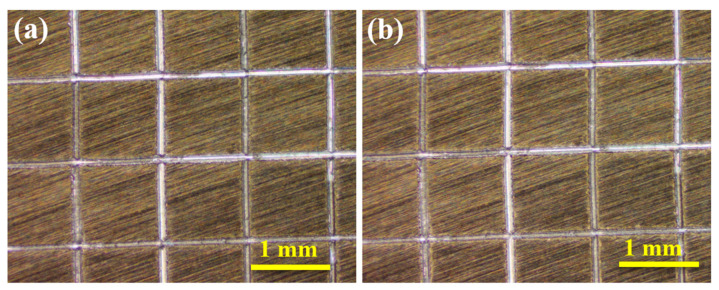
Adhesion test of MgAl-LDH-TTAB film (**a**) before and (**b**) after tape peeling.

Figure 8a,b shows the optical microscope pictures of MgAl-LDH-TTAB film before and after tape peeling. Inspecting the grid area for the removal of coating from the substrate or from a previous coating using the magnifier and the adhesion of coating can be rated in accordance with the detached area. It should be noted that the edges of the cuts are completely the same and no small flakes of the coating are detached at intersections of the squares of the grid area, indicating that the MgAl-LDH-TTAB film has not peeled away from the corners of the substrate or scratch area after tape peeling. Therefore, the MgAl-LDH-TTAB film can be granted by 5A according to the standard of ASTM D3359-97, indicating the superior mechanical adhesion of the film [47].

### 3.4. Corrosion Behaviour of the Studied Samples Determined by PDP

**Figure 9:**
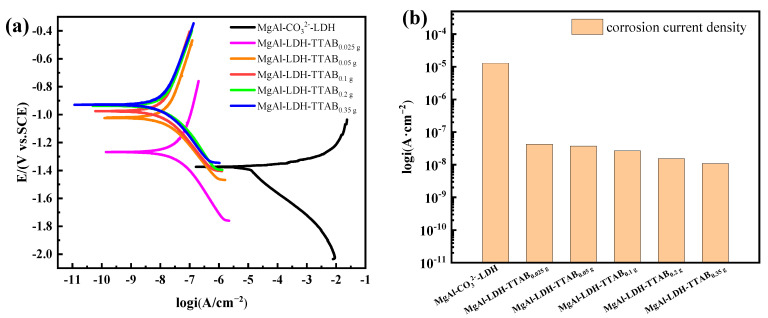
(**a**) PDP curves of MgAl-CO_3_^2−^-LDH film and MgAl-LDH-TTAB_x g_ films in a 3.5 wt.% NaCl solution, (**b**) the corresponding corrosion current density columns.

Figure 9a shows PDP curves of Mg alloys coated with MgAl-CO_3_^2−^-LDH and MgAl-LDH-TTAB_x g_ films in the 3.5 wt.% NaCl solution. The electrochemical parameters obtained from PDP curves are listed in Table 2, where *E_corr_* signifies the corrosion potential, *i_corr_* represents the corrosion current density, and the lower *i_corr_* represents better corrosion resistance.

The *E_corr_* of the MgAl-CO_3_^2−^-LDH film was −1.52 V, whereas the *E_corr_* of the MgAl-LDH-TTAB_0.35 g_ films increased to −0.92 V. The *i_corr_* of the MgAl-CO_3_^2−^-LDH film was 1.29 × 10^−5^ A·cm^−2^ whereas the *i_corr_* of the MgAl-LDH-TTAB_0.35 g_ film was as low as 1.09 × 10^−8^ A·cm^−2^. The more positive *E_corr_* and the lower *i_corr_* of the MgAl-LDH-TTAB film indicated their superior corrosion resistance compared to the MgAl-CO_3_^2−^-LDH film. Additionally, the concentration of TTAB had a great influence on the corrosion performance of MgAl-LDH-TTAB_x g_ films. With the increase of TTAB concentration, the *E_corr_* of the MgAl-LDH-TTAB_x g_ films shifted to the direction of positive potential and the *i_corr_* was decreased. Results illustrated that the corrosion resistance of the MgAl-LDH-TTAB_x g_ films increased with the increase of TTAB concentration. As shown in Table 3, the *i_corr_* of the MgAl-LDH-TTAB film was lower than that of the LDH films previously reported, indicating that LDH films with excellent corrosion resistance can be produced by introducing a surfactant in the process of in situ hydrothermal synthesis.

### 3.5. Corrosion Behaviour of the Studied Samples Determined by EIS

**Figure 10:**
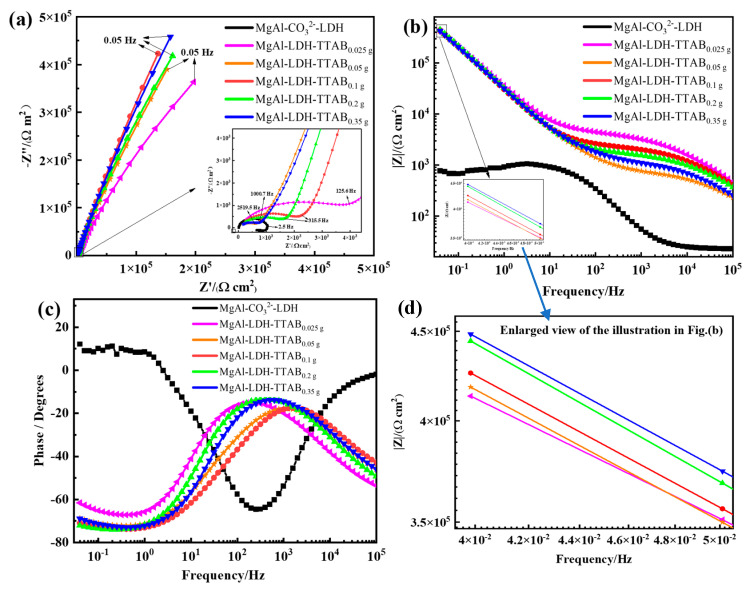
(**a**) Nyquist plots and (**b**,**c**) Bode plots of EIS curves of the MgAl-CO_3_^2−^LDH film and the MgAl-LDH-TTAB_x g_ films, enlarged view (**d**) of the illustration in (**b**).

As shown in Figure 10a, in the Nyquist plots of the MgAl-CO_3_^2−^-LDH film, there is a single impedance semicircle and inductance frequency response behavior. The impedance semicircles represent the coating with medium interface reaction characteristics. The existence of inductance indicates the metal matrix is corroded and the poor MgAl-CO_3_^2−^-LDH film cannot effectively protect the AZ31 substrate submerged in a 3.5 wt.% NaCl solution [50]. As shown in Figure 10a, the semicircle radius in the Nyquist plots of MgAl-LDH-TTAB_x g_ films are much greater than that of the MgAl-CO_3_^2−^-LDH film, indicating that the MgAl-LDH-TTAB_x g_ films have significantly better corrosion resistance than the MgAl-CO_3_^2−^-LDH film. A larger impedance modulus in the low-frequency band in the Bode graphs indicates greater corrosion resistance for an anticorrosion coating [40]. As shown in Figure 10b,d, the |Z|_f = 0.05 Hz_ value of the MgAl-CO_3_^2−^-LDH film is only 7.70 × 10^2^ Ω·cm^2^, which is much lower than that of the MgAl-LDH-TTAB_0.025 g_ film (4.13 × 10^5^ Ω·cm^2^), the MgAl-LDH-TTAB_0.05 g_ film (4.18 × 10^5^ Ω·cm^2^), the MgAl-LDH-TTAB_0.1 g_ film (4.26 × 10^5^ Ω·cm^2^), the MgAl-LDH-TTAB_0.2 g_ film (4.44 × 10^5^ Ω·cm^2^), and the MgAl-LDH-TTAB_0.35 g_ film (4.48 × 10^5^ Ω·cm^2^). These results further confirm that LDH films with excellent corrosion resistance can be produced through the in situ hydrothermal method with the cationic surfactant. Additionally, as shown in Figure 10a,b,d, the semicircle radius in the Nyquist plots and the impedance modulus in the low-frequency of the MgAl-LDH-TTAB_x g_ films increased with the increase of TTAB concentration, which were consistent with the results from polarization curve fitting.

**Figure 11:**
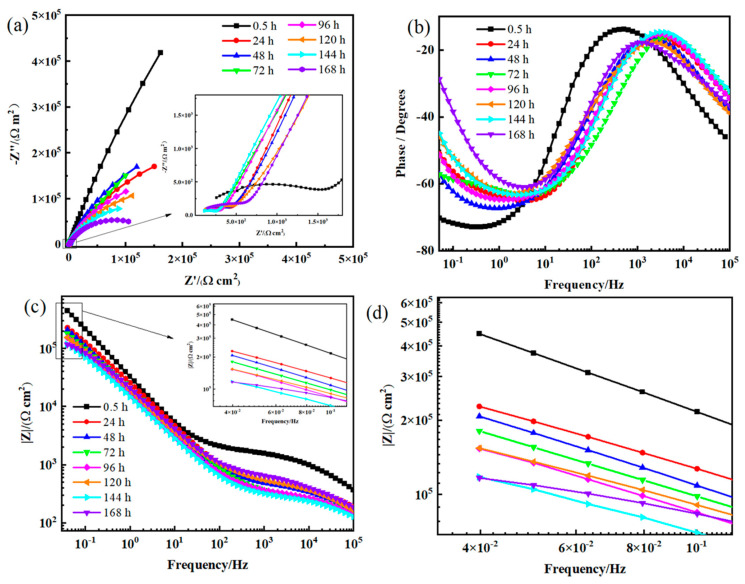
(**a**) Nyquist plots and (**b**,**c**) Bode plots of EIS curves of AZ31 coated with MgAl-LDH-TTAB_0.35 g_ film after different immersion times in 3.5 wt.% NaCl solution, enlarged view (**d**) of the illustration in (**c**).

With a longer time of immersion, the evolution of EIS results was obtained to further indicate the protection performance of the MgAl-LDH-TTAB film on the alloy surface. For the MgAl-LDH-TTAB_0.35 g_ film, the electrochemical behavior underwent a series of variations during the immersion period of 168 h (Figure 11). As shown in Figure 11a, semicircle radius in the Nyquist plots of the MgAl-LDH-TTAB_0.35 g_ film after immersion for 0.5 h were much greater than others, indicating degradation in the protective properties after a long time immersion. Additionally, with the increase of immersion time, the impedance modulus in the low frequency tends to reduce. As shown in Figure 11c,d, |Z|_f = 0.05 Hz_ values of the MgAl-LDH-TTAB_0.35 g_ film were 4.48 × 10^5^ Ω·cm^2^, 2.27 × 10^5^ Ω·cm^2^, 2.07 × 10^5^ Ω·cm^2^, 1.81 × 10^5^ Ω·cm^2^, 1.53 × 10^5^ Ω·cm^2^, 1.54 × 10^5^ Ω·cm^2^, 1.17 × 10^5^ Ω·cm^2^, and 1.16 × 10^5^ Ω·cm^2^, respectively. Reduction of the impedance modulus in the low-frequency band in the Bode graphs indicated the reduction of corrosion resistance for an anticorrosion coating. Although the |Z|_f = 0.05 Hz_ value decreased with the increase of immersion time, it decreased slowly and always maintained a |Z|_f = 0.05 Hz_ value > 10^5^ Ω·cm^2^. These results show that the MgAl-LDH-TTAB_0.35 g_ film still can provide an effective protective effect to the AZ31 substrate after being immersed in 3.5 wt.% NaCl solution for 168 h. Consequently, it is considered that the MgAl-LDH-TTAB_x g_ films produced through the in situ hydrothermal method with a cationic surfactant have good long-term corrosion resistance.

### 3.6. Corrosion Inhibition Mechanism

**Figure 12:**
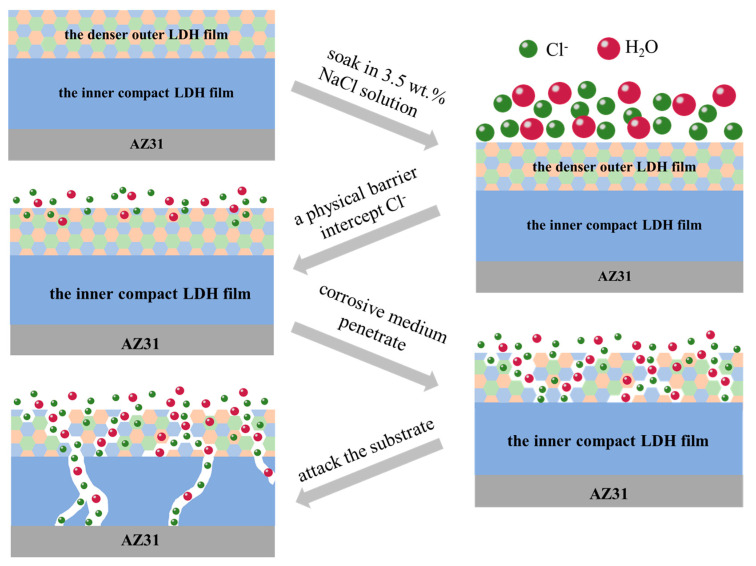
Schematic illustration of the corrosion protection mechanism of MgAl-LDH-TTAB films.

Comparing with MgAl-CO_3_^2−^-LDH film, the *i_corr_* of the MgAl-LDH-TTAB_x g_ film in 3.5 wt.% NaCl solution were reduced 3 order of magnitude (10^−5^ A^.^cm^−2^ to 10^−8^ A^.^cm^−2^), and the |Z|_f = 0.05 Hz_ value was increased 3 order of magnitude (10^2^ Ω·cm^2^ to 10^5^ Ω·cm^2^), respectively. Figure 12 presents the schematic illustration of the structure of the MgAl-LDH-TTAB films for understanding its corrosion protection mechanism. As shown in Figure 12, combined with the results of electrochemical tests and structure characterizations, the high corrosion resistance of the MgAl-LDH-TTAB films on AZ31 is mainly attributed to the following factors: (i) when the AZ31 substrate is soaked in 3.5 wt.% NaCl solution, MgAl-LDH-TTAB films with compact structure can provide an efficient physical barrier to the corrosive medium and avoid direct contact between chloride ions and metal substrates; (ii) MgAl-LDH-TTAB films can intercept Cl^−^ through anion exchange, which minimizes the corrosion of the Mg alloy. Additionally, the better corrosion resistance of the MgAl-LDH-TTAB_x g_ films with the increase of TTAB concentration can be attributed to the denser outer LDH film with the increase of TTAB concentration.

## 4. Conclusions

In this work, MgAl-LDH-TTAB films with high corrosion resistance were produced for the first time using an in situ hydrothermal method with the cationic surfactant TTAB, and the influence of the concentration of TTAB was investigated. The results can be summarized as follows:

(1) MgAl-LDH-TTAB was successfully synthesized in the presence of TTAB, and TTAB was unable to penetrate the LDH layers.
(2) MgAl-LDH-TTAB powders having an average particle size of 100–200 nm were found to be more homogeneous and smaller in particle size than MgAl-CO_3_^2−^-LDH powders.
(3) MgAl-LDH-TTAB films with compact structure, uniform distribution, and superior mechanical adhesion could provide excellent physical shielding from corrosive media. Therefore, MgAl-LDH-TTAB films protected the AZ31 substrate more effectively than the MgAl-CO_3_^2−^-LDH film. The *i_corr_* of the MgAl-LDH-TTAB film was decreased to 1 × 10^−8^ A cm^−2^.
(4) The concentration of TTAB had a significant impact on the morphology and corrosion resistance of LDHs films. With the increase of TTAB concentration, the outer layer LDH films became denser and provided better corrosion resistance for the MgAl-LDH-TTAB films.
(5) After being immersed in 3.5 wt.% NaCl solution for 168 h, the |Z|_f = 0.05 Hz_ values of the AZ31 substrate coated with MgAl-LDH-TTAB_0.35 g_ film still remained at 10^5^ Ω·cm^2^, implying the good long-term corrosion resistance of MgAl-LDH-TTAB_x g_ films.

As a result of this research, adding cationic surfactant in the process of in situ hydrothermal method is considered to be a novel design approach for the production of extremely effective anticorrosion coatings for magnesium alloys.

## Figures and Tables

**Table 1:** The acronyms and the difference of the studied samples.

Sample	Difference	
MgAl-CO_3_^2−^-LDH	obtained from NaOH, Na_2_CO_3,_ magnesium nitrate, and aluminum nitrate	
MgAl-LDH-TTAB_0.025 g_	obtained from NaOH, TTAB, magnesium nitrate, and aluminum nitrate in the N_2_ environment	0.025 g TTAB
MgAl-LDH-TTAB_0.05 g_		0.05 g TTAB
MgAl-LDH-TTAB_0.1 g_		0.1 g TTAB
MgAl-LDH-TTAB_0.2 g_		0.2 g TTAB
MgAl-LDH-TTAB_0.35 g_		0.35 g TTAB

**Table 2:** Electrochemical parameters of AZ31 coated with MgAl-CO_3_^2−^-LDH film and MgAl-LDH-TTAB_x g_ films estimated from the polarization data.

Sample	*E_corr_* (V/SCE)	*i_corr_* (A·cm^−2^)
MgAl-CO_3_^2−^-LDH	−1.52	1.29 × 10^−^^5^
MgAl-LDH-TTAB_0.025 g_	−1.23	4.26 × 10^−8^
MgAl-LDH-TTAB_0.05 g_	−1.02	3.74 × 10^−8^
MgAl-LDH-TTAB_0.1 g_	−0.98	2.66 × 10^−8^
MgAl-LDH-TTAB_0.2 g_	−0.93	1.54 × 10^−8^
MgAl-LDH-TTAB_0.35 g_	−0.92	1.09 × 10^−8^

**Table 3:** Comparison of corrosion resistance of various LDH composite films on the surface of AZ31 (electrolyte: 3.5 wt.% NaCl).

Sample	*E_corr_* (V/SCE)	*i_corr_* (A·cm^−2^)	Reference
MgAl-LDH-TTAB_0.35 g_	−0.92	1.09 × 10^−8^	This study
MgAl-WO_4_^2−^-LDHs	−1.26	7.44 × 10^−6^	[48]
MgAl−8HQ-LDHs	−0.77	1.70 × 10^−7^	[16]
MgAl-ASP-LDHs	0.12	2.24 × 10^−8^	[12]
MgAl-PPA-LDHs	−1.16	2.47 × 10^−9^	[17]
MgAl-NO_3_^−^-LDHs	−1.53	3.10 × 10^−7^	[40]
ZnAl-La-LDHs	−1.07	2.77 × 10^−7^	[49]
ZnAl-ASP-LDHs	−1.50	3.93 × 10^−7^	[49]
ZnAl-MoO_4_^2−^-LDHs	−0.98	3.42 × 10^−6^	[49]
ZnAl-VO_4_^3−^-LDHs	−0.88	3.03 × 10^−7^	[49]

## Data Availability

The data presented in this study are available on reasonable request from the corresponding author.
